# Right Frontal Theta: Is It a Response Biomarker for Ketamine’s Therapeutic Action in Anxiety Disorders?

**DOI:** 10.3389/fnins.2022.900105

**Published:** 2022-07-04

**Authors:** Shabah M. Shadli, Robert G. Delany, Paul Glue, Neil McNaughton

**Affiliations:** ^1^Department of Psychology, University of Otago, Dunedin, New Zealand; ^2^Department of Psychological Medicine, University of Otago, Dunedin, New Zealand

**Keywords:** anxiety disorders, ketamine, electroencephalography (EEG), theta frequency, response biomarker

## Abstract

Anxiety disorders are the most prevalent mental disorders in the world, creating huge economic burdens on health systems and impairing the quality of life for those affected. Recently, ketamine has emerged as an effective anxiolytic even in cases resistant to conventional treatments (TR); but its therapeutic mechanism is unknown. Previous data suggest that ketamine anxiety therapy is mediated by reduced right frontal electroencephalogram (EEG) theta power measured during relaxation. Here we test for a similar theta reduction between population-sample, presumed treatment-sensitive, (TS) anxiety patients and healthy controls. Patients with TS DSM-5 anxiety disorder and healthy controls provided EEG during 10 min of relaxation and completed anxiety-related questionnaires. Frontal delta, theta, alpha1, alpha2, beta, and gamma power, Higuchi’s fractal dimension (HFD) and frontal alpha asymmetry (FAA) values were extracted to match ketamine testing; and we predicted that the controls would have less theta power at F4, relative to the TS anxious patients, and no differences in HFD or FAA. We provide graphical comparisons of our frontal band power patient-control differences with previously published post-pre ketamine TR differences. As predicted, theta power at F4 was significantly lower in controls than patients and FAA was not significantly different. However, HFD was unexpectedly reduced at lateral sites. Gamma power did not increase between controls and patients suggesting that the increased gamma produced by ketamine relates to dissociation rather than therapy. Although preliminary, and indirect, our results suggest that the anxiolytic action of ketamine is mediated through reduced right frontal theta power.

## Introduction

Anxiety disorders are the most prevalent psychiatric diseases in Europe and the United States (Kessler et al., 2005, 2012), the sixth highest in terms of disability (Baxter et al., 2014), and account for about 1/10 suicides (Baxter et al., 2014). They are a grave and ever-increasing burden on healthcare resources (Kessler, 2007; Cryan and Sweeney, 2011; Maron and Nutt, 2017). Most strikingly, anxiety disorders tend to start early in life (Cryan and Sweeney, 2011; Maron and Nutt, 2017) and often result in chronic impairment (Meyer, 2017). In Europe, work days lost because of anxiety are higher than somatic disorders like diabetes (Bandelow and Michaelis, 2015). Across 36 large countries, anxiety and depression are expected to cost 12 billion days every year in lost productivity equivalent to a loss of US$925B (Chisholm et al., 2016). A 1996 survey estimated the cost of anxiety disorders at US$47B in the USA (DuPont et al., 1996; Kessler and Greenberg, 2002). But costs increase every year and, in 2004, the cost for anxiety disorder in Europe was estimated at €41B (Bandelow and Michaelis, 2015) while in 2010, the estimated cost jumped nearly 5-fold to €200B (Olesen et al., 2012; Kalisch et al., 2017). The COVID-19 pandemic significantly increased the number of people diagnosed with anxiety disorders, with recent statistics showing ∼35% of the total population in western societies currently affected (Kowalczyk et al., 2021).

Key problems are that first-line conventional anti-anxiety drugs (which are often also antidepressant) take a long time to act on both anxiety (Bystritsky, 2006; Bandelow et al., 2008) and depression (Muller et al., 2016), improve symptoms for only an unpredictable subset of patients, and fail completely for both anxiety and depression in about 1/3rd of patients (Nemeroff, 2007; Muller et al., 2016). Only some patients respond to the first drug they try, and some do not show any improvement even after trials with multiple drugs (Bystritsky, 2006; Aan Het Rot et al., 2012). First line anxiolytic treatments produce remission in 25–35% and response in 50–60% (Roy-Byrne, 2015), so treatment resistance (TR) is common (Roy-Byrne, 2015; Van Ameringen et al., 2017) and “30–60% of patients have substantial and impairing remaining symptoms” (Bokma et al., 2019). Benzodiazepines are more specific to anxiety (Roy-Byrne, 2015) but have similar TR problems (Cryan and Sweeney, 2011).

Hope is raised by ketamine. A single sub-anesthetic dose of ketamine produces a clear therapeutic response in TR depression within a few hours of administration, which lasts for about a week (Aan Het Rot et al., 2012; Lai et al., 2014; Duman, 2018)—improving mood (Zarate C. A. et al., 2013), reducing suicidal ideation (Duman, 2018), and preventing loss of life (DiazGranados et al., 2010). Previously, we reported that low dose ketamine is also effective in TR anxiety disorder—both generalized (GAD) and social (SAD) (Glue et al., 2017). Ketamine is also effective in OCD (Rodriguez et al., 2013), and PTSD (Feder et al., 2014). Thus, most TR neurotic disorders may respond to ketamine (McNaughton and Glue, 2020).

Unfortunately, we do not know the neural basis for the therapeutic effects of ketamine. Ketamine is most obviously a high potency N-methyl-D-aspartate non-competitive glutamatergic antagonist (Zarate et al., 2006; Aan Het Rot et al., 2012; Murrough et al., 2015; Duman, 2018); but, other NMDA antagonists have not achieved: (1) rapid antidepressant onset; (2) robust efficacy; (3) and sustained efficacy with a single administration (Zarate and Machado-Vieira, 2017). We also recently found no relation between the improvement of anxiety symptoms in TR GAD/SAD and the levels of the ketamine and its metabolites norketmaine (Glue et al., 2019).

However, there are other clues to the basis of ketamine’s therapeutic action. We recently (Shadli et al., 2018) reported effects on relaxation EEG in patients with TR GAD/SAD during ketamine therapy. Ketamine increased high frequency EEG power, and decreased low frequency power. Interestingly, *only the decrease in theta frequency band power at the right frontal site F4* significantly correlated with the rapid changes in anxiety measured by the Fear Questionnaire. These new patient findings appear to fit with earlier preclinical and human data that link anxiolytic action (Shadli et al., 2015) and anxiety disorder (Shadli et al., 2021) to changes in *task-elicited* (as opposed to relaxation) right-frontal theta-band EEG.

The aim of the present study was to assess whether the reported *relaxation* EEG effects of ketamine (Shadli et al., 2018) that correlated with its alleviation of anxiety disorder, match relaxation EEG differences between non-anxious participants and conventional anxiety disorder patients. Shadli et al. (2018), used a variety of other EEG measures to analyze ketamine’s effects. These measures were chosen by them because “in depressed patients, ketamine specifically increases slow wave activity during sleep, especially in those with low baseline slow waves, and this may mediate its antidepressant effects (see Duncan and Zarate, 2013). In healthy participants, it can reduce delta (1–3 Hz), theta (4–7 Hz) and alpha (8–15 Hz) band power, while increasing gamma (> 32 Hz) band power (Hong et al., 2010; de la Salle et al., 2016). But it can also increase theta power while decreasing alpha power (Domino et al., 1965; Schűttler et al., 1987; Kochs et al., 1996), particularly at frontal sites (Muthukumaraswamy et al., 2015); *so changes in bands can be interleaved*, with decreased delta, alpha and beta (16–31 Hz) mixed with increased theta and gamma (Muthukumaraswamy et al., 2015; Rivolta et al., 2015). … [So] we assessed EEG by quantitation of power in specific frequency bands and by measures that show depression-related changes: frontal alpha asymmetry (FAA; Allen et al., 2004; Stewart et al., 2014; Mennella et al., 2017) and increased Higuchi’s fractal dimension (HFD; Higuchi, 1988; Bachmann et al., 2013; Akar et al., 2015).”

Shadli et al. (2018) found no relation between therapeutic action and other power measures, FAA or HFD (Higuchi, 1988). Only F4 theta changes were related to therapeutic effect. Here, we hypothesized that healthy controls will have lower power in the theta band at the F4 channel than the anxiety disorder patients (but did not exclude other power changes). We also hypothesized that anxiety disorder patients and controls would not differ on FAA or HFD scores.

## Materials and Methods

There were 34 (26 female, 8 male) healthy and 47 (39 female, 8 male) patient participants recruited through online advertisements on a local newspaper site, Facebook, and advertisements in supermarkets. Healthy participants reported no major illness in the past month, were not prescribed any psychoactive medication in the previous 6 months, and had not consumed alcohol in the 24 h prior to participating in the study. The patient group consisted of individuals who reported experiencing chronic symptoms of anxiety or fear, but were not receiving any pharmacological treatments at the time of recruitment. Participants were also excluded from this study if they had any history of substance abuse or other neurological disorders. All patients (29 GAD, 10 SAD, and 8 PTSD) went through the Mini International Neuropsychiatric Interview (MINI) diagnostic examination by a clinical psychologist in a separate session before having their electroencephalogram (EEG) recorded. Similar to the control group, these participants were otherwise healthy. They reported no significant illness in the past month, no use of psychoactive medications in the previous 6 months, and no consumption of alcohol in the 24 h before the experiment. All participants received petrol vouchers ($30) in compensation for their time and travel costs. The study was approved by the University of Otago Ethics Committee (Health: H15/005), and all participants provided written informed consent.

### Questionnaires and Demographics

To avoid questionnaire fatigue, the questionnaires were administered in two sets. The first set of questionnaires was administered before EEG recording: the Spielberger State-Trait Anxiety Inventory form-Y (Spielberger et al., 1983); the Eysenck Personality Questionnaire-Revised (EPQ-R; Eysenck and Eysenck, 1991); and the BIS scale items from the Behavioral Activation System/Behavioral Inhibition System questionnaire (Carver and White, 1994). The second set was administered after the EEG recording, and contained a subset of scales from the Personality Inventory of the DSM-5 (PID-5) (Anderson et al., 2013). Table 1 represents the demographic details of patients and healthy volunteers.

**TABLE 1:** shows the mean and *SD* for age, STAI-T (T), EPQ Neuroticism (N), PID-5 anxiety (Ax), anhedonia (Ah), and depression (D) scores for each of patients and healthy controls.

	Age		T		N		Ax		Ah		D	
Patients	33.3	*10.5*	53.1	*10.6*	14.5	*5.5*	28.4	*5.7*	17.1	*4.7*	29.6	*9.1*
Controls	31.0	*6.4*	36.3	*4.7*	4.6	*5.8*	16.6	*8.4*	12.8	*5.6*	20.5	*9.4*

### Electroencephalogram Recording

EEG data were recorded using a 32-channel Waveguard EEG cap (ANT Neurotechnology, Netherlands). The electrodes on the cap were arranged in accordance with to the 10–20 electrode placement system. EEG was recorded, sampled at 512 Hz, from 32 channels: Fp1, Fpz, Fp2, F7, F3, Fz, F4, F8, FC5, FC1, FC2, FC6, T7, C3, Cz, C4, T8, CP5, CP1, CP6, CP2, P7, P3, Pz, P4, P8, POz, Oz, O1, O2, M1 and M2 with CPz used as recording reference. Only the frontal electrodes F7, F3, Fz, F4, and F8 were analyzed to compare with our previous ketamine findings. The EEG was re-referenced to the average of M1 + M2 for analysis. Electro-gel (Electro Cap International, United States) was injected into all electrodes using a 3 ml syringe and a Precision Glide 16-gauge blunt needle (Becton, Dickenson & Co., New Jersey, United States). Impedance was brought down to below 20 KΩ for every electrode. 10 min of Resting EEG data were recorded in 1-min blocks of eyes open (EO) or eyes closed (EC) in the following sequence: EO, EC, EO, EC, EO, EC, EO, EC, EO, EC.

### Data Processing and Analysis

#### Primary Pre-processing

We used the same EEG post-processing as our previous experiment (Shadli et al., 2018). EEG data and associated event markers were imported to the EEGLAB toolbox for MATLAB. Raw data were first down sampled to 128 Hz then a 1–63 Hz bandpass filter was applied. 50 Hz noise was removed using Cleanline (Bigdely-Shamlo et al., 2015). Data sets were epoched as 1 s (128 samples) non-overlapping epochs for automatic artifact rejection. Epoched data sets were visually inspected for gross artifacts and removed from the dataset and boundary markers were inserted to mark their previous locations. Independent component analysis (ICA) was subsequently applied to the remaining epoched data. ADJUST 1.1 (Mognon et al., 2011) was used to analyze the ICA results and remove artifact components to leave “clean” EEG. Artifact-free datasets were subsequently converted from epoched to continuous. Similar to our previous experiment (Shadli et al., 2018), we analyzed FAA and HFD.

#### Spectral Analysis

Artifact free datasets were re-epoched to 2 s, 50% overlapping, epochs with a Hanning window. A fast Fourier transform was applied, and the power spectrum was log_10_ transformed to normalize error variance. The resultant epochs were averaged to provide a single power spectrum for each participant, at each channel, and frequency values were averaged in bands defined as delta (1–3 Hz), theta (4–6 Hz), alpha1 (7–9 Hz), alpha2 (10–12 Hz), beta (25–34 Hz), and gamma (41–53 Hz) as previously (Shadli et al., 2018). FAA was calculated for 7–12 Hz by subtracting logarithmic power at left electrodes from their right-most counterparts [(ln (R)–ln (L)] for each of F8:F7 and F4:F3. This was for the purpose of directly comparing to the FAA results of Shadli et al. (2018).

“Fractal dimension was calculated using Higuchi’s algorithm with a kmax of 8 (Higuchi, 1988). After the eye-blink removal stage, the data were subjected to an additional 2–36-Hz bandpass filter, and sections with artefacts were manually removed. The continuous data were then split into 2-s (256 sample) epochs with 50% overlap. Higuchi’s algorithm creates kmax number of new time series (with k running from 1 to kmax), each obtained by taking every kth sample of the original epoch. The length of the curve of each series is calculated and plotted against k on a double logarithmic graph. If the length of the curve and k are proportional, then the plotted data will fall on a straight line. The slope of this line is the fractal dimension.” (Shadli et al., 2018, p. 719).

### Statistical Analysis

Statistical analysis was carried out with IBM SPSS (version 24). Mixed measures ANOVAs with group (patients, controls) as a between-subjects factor were carried out on each of band power, FAA, and HFD. For band power, channel (F7, F3, Fz, F4, and F8), and band (delta, theta, alpha1, alpha1, beta and gamma) were repeated measures with orthogonal polynomial components of channel and band automatically extracted by SPSS. For each band, log power values at each frequency were averaged to a single value prior to ANOVA. HFD was analyzed similarly except for there being no band factor. For FAA, asymmetry was calculated separately for the F7:F8 pair and the F3:F4 pair, and the two values treated as levels of a repeated measures factor “electrode pair.” Significant effects were further explored where necessary with *post-hoc t*-tests.

## Results

### Patient vs. Control Overview

**FIGURE 1:**
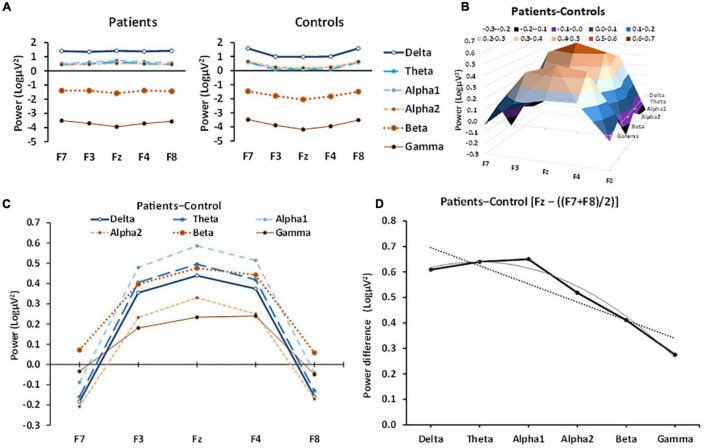
Patient and control EEG power in the different bands across frontal channels. **(A)** Patient power curves are relatively flat across each channel for most bands with the possible exception of gamma, while control power curves generally have an inverted U-shape. **(B)** 3D representation of the effect of anxiety (patient-control power difference) in different bands at frontal-central electrodes, showing the systematic left-right channel × band frequency variation, detected statistically by a significant channel[quadratic] × frequency[linear] interaction. **(C)** The same data as in **B** but with different bands plotted against channel as overlapping curves to allow numerical comparison (frequency of the band is coded by line thickness). The anxiety effect for each band was an inverted-U function of channel; that is, the strongest difference in power was in the central channel, with minimal anxiety effects in the lateral channels. **(D)** Linear trend and quadratic trend of band frequency fitted to power values for Fz minus the average of F7 + F8 (which approximates the quadratic trend across channel). There is a significant decrease in this component from delta to gamma (the dotted line shows the significant linear trend). There appears to be some non-linearity, with an apparent peak at alpha1. The curved line shows the combination of the linear trend with the apparent slight, non-significant (*p* = 6%), quadratic trend.

Figure 1A displays the separate patient group and control group band power values across frontal channels (F7, F3, Fz, F4, and F8). Patients had largely similar power across channels. As band frequency increased, power decreased, with delta expressing the highest power and gamma the lowest. Theta, alpha1 and alpha2 power were all approximately equal across all channels. Control band frequency is also inversely proportional to power, with higher frequency bands displaying lower power values. However, controls displayed an inverted-U distribution of power across channels, with power in the central channel (Fz) lower than in lateral channels (F7, F8).

### Anxiety Effect

To allow a clearer picture of the role of anxiety, the patient-control *difference* is plotted in Figure 1B and shows the 3D relationship between frontal channel position and band frequency. The same data are plotted, overlayed, in Figure 1C to allow direct numerical comparison of the power bands and shows that in anxiety patients power increased at the midline (Fz), relative to control patients across *all* frequency bands. In the lateral channels (F7, F8), the difference between control and anxiety patient power was minimal and usually a decrease. Thus, the effect of anxiety on band power produced an inverted U-shape curve that varied in size, systematically (Figures 1C,D). This change was largely a progressive decrease from delta through gamma [group × band[***lin***] × left-right[quad], *F*(1, 79) = 10.021, *p* = 0.002] but with a marginal inflection with lower values either side of alpha [group × band[***quad***] × left-right[quad], *F*(1, 79) = 3.573, *p* = 0.062]. The linear and quadratic frequency trends of the inverted-U variation in the anxiety effect can be seen in a simplified form by plotting power for Fz minus the average power of F7 and F8 as a proxy for the quadratic trend of channel (Figure 1D). There is a predominantly linear fall off with frequency in the average anxiety effect (dotted straight line) except that the effect for delta is below what would be expected for a purely linear effect (solid gray linear + quadratic curve).

### Frontal Band Power—Qualitative Comparison With Ketamine

**FIGURE 2:**
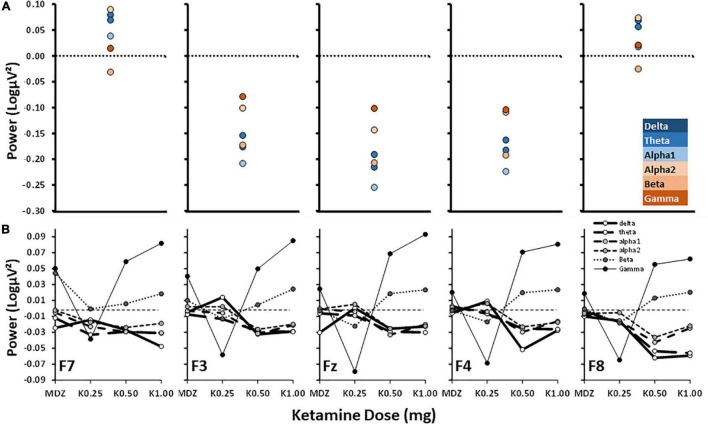
Power difference scores for control–patient and post–pre ketamine across bands and frontal channels. **(A)** Control–patient power is as in Figure 1C but with the direction of subtraction reversed and graphs plotted to match the published results shown below in **(B)**. **(B)** Post–pre dose effect of ketamine from Shadli et al. (2018) with permission of the authors. MDZ = midazolam (active control); K0.25, etc., doses of ketamine in mg. Note power scale differences.

Figure 2 compares the current “anxiolytic” effects on frontal channel band power (Figure 2A) with those obtained with various doses of ketamine (Figure 2B, adapted from Shadli et al., 2018). Patient power values were subtracted from control power values to mimic ketamine’s anxiolytic effect (i.e., the opposite of the subtraction in Figure 1) matching the post–pre subtraction used by Shadli et al. (2018).

Figure 2A shows that control—anxiety patient power differences were distributed from F7 to F8 in a U-shaped curve, with the effect at lateral channels being close to zero. However, non-anxious power at more central channels was reduced compared to anxious patients. Across the central channels, alpha1 power showed the largest decrease and gamma expressed the highest increase.

Figure 2B shows the published effects of varying doses of post-ketamine administration relative to pre-ketamine administration (post-pre difference) in power across frontal channels. The post—pre effects increased steadily with dose across all channels: unlike Figure 2A, high frequency power increased (with beta and, particularly, gamma); while, like Figure 2A, low frequency power decreased (delta, theta, alpha1 and alpha2). So, in general, the effects of ketamine are opposite to the control-patient difference at high frequencies but similar at low frequencies.

### Higuchi’s Fractal Dimension and Frontal Alpha Asymmetry Comparison

**FIGURE 3:**
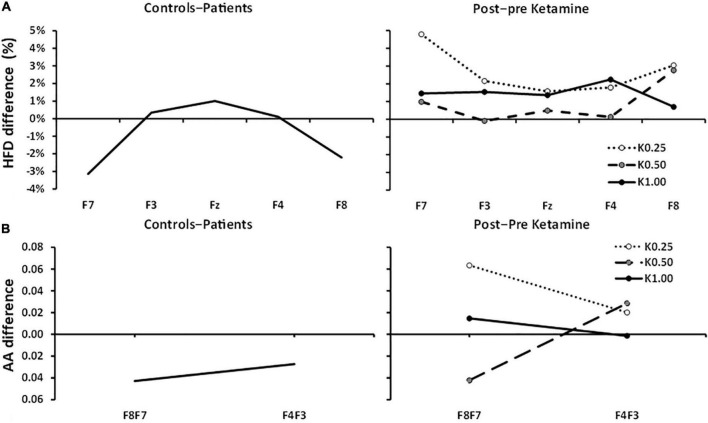
Controls–patients and post–pre ketamine HFD and AA differences across frontal channels. **(A)** HFD: Controls–patients displays a significant U-shaped relationship between HFD change and frontal channels. 0.25, 0.5, and 1.0 mg/kg of ketamine produced no significant HFD effects and any trend is in the opposite direction. **(B)** Controls–Patients FAA differences were not significant at F8:F7 and F4:F3 pairs. Post–pre ketamine FAA was not significantly different across 0.25, 0.50, and 1.00 mg/kg doses at F8:F7 and F4:F3 pairs.

Figure 3A displays the anxiety *reduction* effect (controls–patients) on HFD and compares this to the post*-*pre ketamine HFD scores of Shadli et al. (2018). There was a clear inverted-U difference between controls and patients with the lateral channels showing larger negative values [group × channel[quad], *F*(1, 79) = 6.680, *p* = 0.012]. There was little difference in HFD at Fz [*t*(79) = 0.534, NS]. There were no significant differences in HFD reported by Shadli et al. (2018). Further, K0.50 produced the highest decrease in Fear Questionnaire scores but minimal change in HFD. The non-significant quadratic trends with K0.25 and K1.00 are in the opposite direction to the current results.

FAA, averaged across electrode pair and group, was not significantly different from zero [intercept, *F*(1, 79) = 1.181, *p* = 0.281]. Figure 3B shows the FAA control*-*patient differences. FAA, averaged across electrode pair, did not differ between controls and patients [group, *F*(1, 79) = 1.682, *p* = 0.198] and showed no differences between the electrode pairs in the control*-*patient differences [electrode pair × group, *F*(1, 79) = 0.150, *p* = 0.700].

## Discussion

### Overview of Findings

Our primary finding, *using identical procedures to our previous work with ketamine*, was that patients diagnosed with GAD, SAD, SP, and PTSD showed increases in frontal relaxation EEG rhythmicity compared to the controls. There was no anxiety effect at the lateral channels but anxiety was associated with significantly higher power centrally, across all frequency bands. These data are consistent with earlier findings with panic disorder patients compared to healthy controls (Wise et al., 2011). At Fz, power difference was generally inversely proportional to frequency, with a modest peak in the alpha range. As predicted, AA was unaffected and, while there was a significant effect on HFD at lateral channels, this was in the opposite direction to the non-significant trends in the ketamine data.

### Comparison With Ketamine Study

#### Theta Reduction at F4

Our primary predicted finding was that control theta power decreased at F4 relative to the patients. Both here, and in Shadli et al. (2018), theta reduced at several frontal channels with reduced anxiety. However, only F4 theta reduction predicted anxiety symptom improvements with ketamine as measured by the Fear Questionnaire. Our F4 result, therefore, supports the notion that the anxiolytic action of ketamine might be mediated through reduced theta power in the F4 channel.

We also found stronger theta at Fz in patients relative to healthy controls and no differences at the lateral channels (F7 and F8)—consistent with a recent review concluding that midline theta is an important index of psychiatric illness (McLoughlin et al., 2022). Frontal midline theta is a distinct rhythm recorded at Fz above the midcingulate cortex (Mitchell et al., 2008). It lasts a few seconds during arithmetic mental tasks, such as addition in the N-back working memory task; and has since been associated with anxiety-like behavior. However, there are several forms of such theta rhythmicity. Administration of anxiolytic drugs, including benzodiazepines and buspirone, *increase* frontal-midline theta power in the Uchida-Kraepelin task with associated decreases in STAI (anxiety) scores (for review see Mitchell et al., 2008).

The apparent specificity of the involvement of F4 is unexpected. The changes linked to psychiatric disorder in right frontal theta (Shadli et al., 2018) and in the distinct midfrontal theta (Mitchell et al., 2008; McLoughlin et al., 2022) appear more widespread—consistent with our present results. However, source separation has demonstrated multiple midfrontal thetas (Zuure et al., 2020) and these appear involved a range of types of cognitive control (Cavanagh and Frank, 2014). It is likely, then, that ketamine’s wide-ranging effects (both across brain sites and across frequency bands) are the result of impact on multiple mechanisms, the bulk of which reflect longer-term consequences of anxiety (e.g., changes in theta) or immediate side-effects (e.g., changes in gamma, see below). The F4 effect, then, would be related to a specific mechanism that, rather than being a consequence, is causally related to the generation or maintenance of anxiety. Much further work would be needed to determine if this is the case.

The mechanisms of action of ketamine on psychiatric disorders in general and anxiety and F4 theta in particular are unclear. As noted in the introduction, its therapeutic effect is not *via* its known NMDA receptor effects (Zarate et al., 2006; Aan Het Rot et al., 2012; Murrough et al., 2015; Zarate and Machado-Vieira, 2017; Duman, 2018) nor linked to its metabolites (Glue et al., 2019). Given its wide-ranging effects on brain activity, most of which appear causally unrelated to anxiety it will be hard to uncover its primary therapeutic mechanisms. Its effects on theta rhythmicity (Engin et al., 2009) in a well-validated rat electrophysiology model of anxiolytic action (McNaughton et al., 2007) could provide a guide to its anxiolytic action. Whether its antidepressant actions are related to theta (or F4) remains to be determined but, given the nature of the drugs detected by the rat model, is unlikely.

#### Gamma Band Changes

With ketamine (Shadli et al., 2018), gamma power across frontal channels (including F4) increased. In contrast, gamma power at F4 in control participants relative to anxiety disorder patients decreased. This implies that the increase in gamma power after ketamine administration is unrelated to its anxiolytic action. At the doses used in our previous studies of anxiety reduction, ketamine produces strong dissociative effects (Glue et al., 2019), which include euphoria and hallucinations. Since these are not symptoms of anxiety it suggests that an increase in gamma power is associated with the hallucinatory effects of ketamine. Gamma frequency has previously been linked to NMDA antagonism. Blockade of the NMDA receptors in rat neocortex *in vivo* has led to dose-dependent increase in gamma power (Pinault, 2008). Administration of NMDA receptor antagonists, such as ketamine, have also induced hallucinations in healthy participants (Krystal et al., 1994; Lahti et al., 1995). These studies imply that the dissociative effects of ketamine result from its NMDA antagonist properties, causing an increase in gamma band power.

#### Higuchi’s Fractal Dimension Changes

Shadli et al. (2018) found no significant HFD differences between pre- and post-ketamine administration at any dosage. In contrary, we found significant differences in HFD at the lateral channels (F7 and F8) between anxiety patients and controls, consistent with our earlier findings (Kawe et al., 2019). These results suggest that a shift from an anxious to a non-anxious state is related to a reduction in HFD at the lateral channels, corresponding to left and right prefrontal sites. Note that the direction of change here is the opposite of the non-significant trend differences with ketamine. The HFD differences, here, are also at lateral sites whereas the theta power differences are not.

#### Alpha Asymmetry Differences

No significant difference was observed in FAA between anxiety disorder patients and controls—consistent with our hypothesis that no difference in FAA would be observed between anxiety patients and controls. Shadli et al. (2018) found no significant difference between pre-ketamine and post-ketamine treatment on FAA at any dose.

### Overview of Findings and Limitations

There is an urgent need to uncover the causes of anxiety disorders to reduce the global crisis, which is being worsened by the COVID-19 pandemic. Ketamine and similar fast acting drugs could be a game changer in the treatment of anxiety disorders. However, ketamine is currently an off-label drug because of its abuse potential, and strong dissociative and hallucination effects, limiting its use out of the clinic. Continuous efforts are needed to better understand its mechanism of action, which will provide for development of similar drugs with reduced side effects. Given our low sample size, our findings need to be approached with considerable caution. Future studies should have larger sample sizes of both anxious and healthy volunteers and more direct comparison of ketamine affects with natural anxiety differences. Further our method is comparative and while consistent with our hypothesis and strengthening our previous conclusions about ketamine, cannot be taken as proof. That said, our *right frontal theta (F4)* or the quite distinct frontal-central (Fz) theta should provide guidance toward better understanding of the mechanism of action of ketamine in anxiety disorders.

## Data Availability Statement

The raw data supporting the conclusions of this article will be made available by the authors, on reasonable request, without undue reservation.

## Ethics Statement

The studies involving human participants were reviewed and approved by the University of Otago Human Ethics Committee. The patients/participants provided their written informed consent to participate in this study.

## Author Contributions

SS and NM developed the research idea. SS and RD collected and processed the data. PG screened and consented patient volunteers. SS wrote up the initial draft. NM and PG revised the manuscript. All authors contributed to the article and approved the submitted version.

## Conflict of Interest

PG has a contract with Douglas Pharmaceuticals to develop novel ketamine formulations. The remaining authors declare that the research was conducted in the absence of any commercial or financial relationships that could be construed as a potential conflict of interest.

## Publisher’s Note

All claims expressed in this article are solely those of the authors and do not necessarily represent those of their affiliated organizations, or those of the publisher, the editors and the reviewers. Any product that may be evaluated in this article, or claim that may be made by its manufacturer, is not guaranteed or endorsed by the publisher.

## References

[B1] Aan Het Rot M. Zarate C. A. Jr. Charney D. S. Mathew S. J. (2012). Ketamine for depression: where do we go from here? *Biol. Psychiatry* 72 537–547. 10.1016/j.biopsych.2012.05.003 22705040 PMC3438349

[B2] Akar A. S. Kara S. Agambayev S. Bilgic V. (2015). Nonlinear analysis of EEGs of patients with major depression during different emotional states. *Comput. Biol. Med.* 67 49–60. 10.1016/j.compbiomed.2015.09.019 26496702

[B3] Allen J. J. Urry H. L. Hitt S. K. Coan J. A. (2004). The stability of resting frontal electroencephalographic asymmetry in depression. *Psychophysiology* 41 269–280. 10.1111/j.1469-8986.2003.00149.x 15032992

[B4] Anderson J. L. Sellbom M. Bagby R. M. Quilty L. C. Veltri C. O. Markon K. E. (2013). On the convergence between PSY-5 domains and PID-5 domains and facets: implications for assessment of DSM-5 personality traits. *Assessment* 20 286–294. 10.1177/1073191112471141 23297369

[B5] Bachmann M. Lass J. Suhhova A. Hinrikus H. (2013). Spectral asymmetry and Higuchi’s fractal dimension measures of depression electroencephalogram. *Comput. Math. Methods Med.* 2013:251638. 10.1155/2013/251638 24232245 PMC3819823

[B6] Bandelow B. Michaelis S. (2015). Epidemiology of anxiety disorders in the 21st century. *Dialogues Clin. Neurosci.* 17 327–335. 26487813 10.31887/DCNS.2015.17.3/bbandelow PMC4610617

[B7] Bandelow B. Zohar J. Hollander E. Kasper S. Moller H. J. Post-Traumatic Stress, et al. (2008). World Federation of Societies of Biological Psychiatry (WFSBP) guidelines for the pharmacological treatment of anxiety, obsessive-compulsive and post-traumatic stress disorders – first revision. *World J. Biol. Psychiatry* 9 248–312. 10.1080/15622970802465807 18949648

[B8] Baxter A. J. Vos T. Scott K. M. Ferrari A. J. Whiteford H. A. (2014). The global burden of anxiety disorders in 2010. *Psychol. Med.* 44 2363–2374. 10.1017/S0033291713003243 24451993

[B9] Bigdely-Shamlo N. Mullen T. Kothe C. Su K.-M. Robbins K. A. (2015). The PREP pipeline: standardized preprocessing for large-scale EEG analysis. *Front. Neuroinform.* 9:16. 10.3389/fninf.2015.00016 26150785 PMC4471356

[B10] Bokma W. A. Wetzer G. Gehrels J. B. Penninx B. Batelaan N. M. van Balkom A. (2019). Aligning the many definitions of treatment resistance in anxiety disorders: a systematic review. *Depress. Anxiety* 36 801–812. 10.1002/da.22895 31231925 PMC6771798

[B11] Bystritsky A. (2006). Treatment-resistant anxiety disorders. *Mol. Psychiatry* 11 805–814. 10.1038/sj.mp.4001852 16847460

[B12] Carver C. S. White T. L. (1994). Behavioral inhibition, behavioral activation, and affective responses to impending reward and punishment: the BIS/BAS scales. *J. Pers. Soc. Psychol.* 67 319–333.

[B13] Cavanagh J. F. Frank M. J. (2014). Frontal theta as a mechanism for cognitive control. *Trends Cogn. Sci.* 18 414–421. 10.1016/j.tics.2014.04.012 24835663 PMC4112145

[B14] Chisholm D. Sweeny K. Sheehan P. Rasmussen B. Smit F. Cuijpers P. (2016). Scaling-up treatment of depression and anxiety: a global return on investment analysis. *Lancet Psychiatry* 3 415–424. 10.1016/s2215-0366(16)30024-4 27083119

[B15] Cryan J. F. Sweeney F. F. (2011). The age of anxiety: role of animal models of anxiolytic action in drug discovery. *Br. J. Pharmacol.* 164 1129–1161. 10.1111/bph.2011.164.issue-4 21545412 PMC3229755

[B16] de la Salle S. Choueiry J. Shah D. Bowers H. McIntosh J. Ilivitsky V. (2016). Effects of ketamine on resting-state EEG activity and their relationship to perceptual/dissociative symptoms in healthy humans. *Front. Pharmacol.* 7:348. 10.3389/fphar.2016.00348 27729865 PMC5037139

[B17] DiazGranados N. Ibrahim L. A. Brutsche N. E. Ameli R. Henter I. D. Luckenbaugh D. A. (2010). Rapid resolution of suicidal ideation after a single infusion of an N-methyl-D-aspartate antagonist in patients with treatment-resistant major depressive disorder. *J. Clin. Psychiatry* 71 1605–1611. 10.4088/JCP.09m05327blu 20673547 PMC3012738

[B18] Domino E. F. Chodoff P. Corssen G. (1965). Pharmacologic effects of CI-581, a new dissociative anesthetic, in man. *Clin. Pharmacol. Ther.* 6 279–291. 10.1002/cpt196563279 14296024

[B19] Duman R. S. (2018). Ketamine and rapid-acting antidepressants: a new era in the battle against depression and suicide. *F1000 Res.* 7:e659. 10.12688/f1000research.14344.1 29899972 PMC5968361

[B20] Duncan W. C. Jr. Zarate C. A. Jr. (2013). Ketamine, sleep, and depression: current status and new questions. *Curr. Psychiatry Rep.* 15:394. 10.1007/s11920-013-0394-z 23949569 PMC3827949

[B21] DuPont R. L. Rice D. P. Miller L. S. Shiraki S. S. Rowland C. R. Hanvood H. J. (1996). Economic cost of anxiety disorder. *Anxiety* 2 167–172. 9160618 10.1002/(SICI)1522-7154(1996)2:4<167::AID-ANXI2>3.0.CO;2-L

[B22] Eysenck H. J. Eysenck S. B. G. (1991). *ADULT EPQ-R.* London: Hodder Education.

[B23] Engin E. Treit D. Dickson C. T. (2009). Anxiolytic- and antidepressant-like properties of ketamine in behavioral and neurophysiological animal models. *Neuroscience* 161 359–369. 10.1016/j.neuroscience.2009.03.038 19321151

[B24] Feder A. Parides M. K. Murrough J. W. Perez A. M. Morgan J. E. Saxena S. (2014). Efficacy of intravenous ketamine for treatment of chronic posttraumatic stress disorder: a randomized clinical trial. *JAMA Psychiatry* 71 681–688. 10.1001/jamapsychiatry.2014.62 24740528

[B25] Glue P. Medlicott N. J. Harland S. Neehoff S. Anderson-Fahey B. Le Nedelec M. (2017). Ketamine’s dose-related effects on anxiety symptoms in patients with treatment refractory anxiety disorders. *J. Psychopharmacol.* 31 1302–1305. 10.1177/0269881117705089 28441895

[B26] Glue P. Neehoff S. Sabadel A. Broughton L. Le Nedelec M. Shadli S. (2019). Effects of ketamine in patients with treatment-refractory generalized anxiety and social anxiety disorders: exploratory double-blind psychoactive-controlled replication study. *J. Psychopharmacol.* 34 267–272. 10.1177/0269881119874457 31526207

[B27] Higuchi T. (1988). Approach to an irregular time series on the basis of the fractal theory. *Phys. D* 31 277–283.

[B28] Hong L. E. Summerfelt A. Buchanan R. W. O’Donnell P. Thaker G. K. Weiler M. A. (2010). Gamma and delta neural oscillations and association with clinical symptoms under subanesthetic ketamine. *Neuropsychopharmacology* 35 632–640. 10.1038/npp.2009.168 19890262 PMC3055615

[B29] Kalisch R. Baker D. G. Basten U. Boks M. P. Bonanno G. A. Brummelman E. (2017). The resilience framework as a strategy to combat stress-related disorders. *Nat. Hum. Behav.* 1 784–790. 10.1038/s41562-017-0200-8 31024125

[B30] Kawe T. N. J. Shadli S. M. McNaughton N. (2019). Higuchi’s fractal dimension, but not frontal or posterior alpha asymmetry, predicts PID-5 anxiousness more than depressivity. *Sci. Rep.* 9:19666. 10.1038/s41598-019-56229-w 31873184 PMC6928148

[B31] Kessler R. C. (2007). The global burden of anxiety and mood disorders: putting ESEMeD findings into perspective. *J. Clin. Psychiatry* 68 10–19. PMC1852440 17288502

[B32] Kessler R. C. Greenberg P. E. (2002). “The economic burden of anxiety and stress disorders,” in *Neuropsychopharmacology: The Fifth Generation of Progress*, eds Kenneth D. C. Davis L. Coyle J. T. Nemeroff C. (Baltimore, MD: American College of Neuropsychopharmacology).

[B33] Kessler R. C. Chiu W. T. Demler O. Merikangas K. R. Walters E. E. (2005). Prevalence, severity, and comorbidity of 12-month DSM-IV disorders in the National Comorbidity Survey Replication. *Arch. Gen. Psychiatry* 62 617–627. 10.1001/archpsyc.62.6.617 15939839 PMC2847357

[B34] Kessler R. C. Petukhova M. Sampson N. A. Zaslavsky A. M. Wittchen H. U. (2012). Twelve-month and lifetime prevalence and lifetime morbid risk of anxiety and mood disorders in the United States. *Int. J. Methods Psychiatr. Res.* 21 169–184. 10.1002/mpr.1359 22865617 PMC4005415

[B35] Kochs E. Scharein E. Möllenberg O. Bromm B. Schulte am Esch J. (1996). Analgesic efficacy of low-dose ketamine. *Anesthesiology* 85 304–314. 8712446 10.1097/00000542-199608000-00012

[B36] Kowalczyk M. Kowalczyk E. Kwiatkowski P. Lopusiewicz L. Sienkiewicz M. Talarowska M. (2021). Ketamine-new possibilities in the treatment of depression: a narrative review. *Life* 11:1186. 10.3390/life11111186 34833062 PMC8619908

[B37] Krystal J. H. Karper L. P. Seibyl J. P. Freeman G. K. Delaney R. Bremner J. D. (1994). Subanesthetic effects of the noncompetitive NMDA antagonist, ketamine, in humans: psychotomimetic, perceptual, cognitive, and neuroendocrine responses. *Arch. Gen. Psychiatry* 51 199–214. 10.1001/archpsyc.1994.03950030035004 8122957

[B38] Lahti A. C. Koffel B. LaPorte D. Tamminga C. A. (1995). Subanesthetic doses of ketamine stimulate psychosis in schizophrenia. *Neuropsychopharmacology* 13 9–19. 8526975 10.1016/0893-133X(94)00131-I

[B39] Lai R. Katalinic N. Glue P. Somogyi A. A. Mitchell P. B. Leyden J. (2014). Pilot dose-response trial of i.v. ketamine in treatment-resistant depression. *World J. Biol. Psychiatry* 15 579–584. 10.3109/15622975.2014.922697 24910102

[B40] Maron E. Nutt D. (2017). Biological markers of generalized anxiety disorder. *Dialogues Clin. Neurosci.* 19 147–157. 28867939 10.31887/DCNS.2017.19.2/dnutt PMC5573559

[B41] McLoughlin G. Gyurkovics M. Palmer J. Makeig S. (2022). Midfrontal theta activity in psychiatric illness: an index of cognitive vulnerabilities across disorders. *Biol. Psychiatry* 91 173–182. 10.1016/j.biopsych.2021.08.020 34756560

[B42] McNaughton N. Glue P. (2020). Ketamine and neuroticism: a double-hit hypothesis of internalizing disorders. *Pers. Neurosci.* 3:e2. 10.1017/pen.2020.2 32524063 PMC7253687

[B43] McNaughton N. Kocsis B. Hajós M. (2007). Elicited hippocampal theta rhythm: a screen for anxiolytic and procognitive drugs through changes in hippocampal function? *Behav. Pharmacol.* 18 329–346. 10.1097/FBP.0b013e3282ee82e3 17762505

[B44] Mennella R. Patron E. Palomba D. (2017). Frontal alpha asymmetry neurofeedback for the reduction of negative affect and anxiety. *Behav. Res. Ther.* 92 32–40. 10.1016/j.brat.2017.02.002 28236680

[B45] Meyer A. (2017). A biomarker of anxiety in children and adolescents: a review focusing on the error-related negativity (ERN) and anxiety across development. *Dev. Cogn. Neurosci.* 27 58–68. 10.1016/j.dcn.2017.08.001 28818707 PMC6987910

[B46] Mitchell D. J. McNaughton N. Flanagan D. Kirk I. J. (2008). Frontal-midline theta from the perspective of hippocampal “theta”. *Prog. Neurobiol.* 86 156–185. 10.1016/j.pneurobio.2008.09.005 18824212

[B47] Mognon A. Jovicich J. Bruzzone L. Buiatti M. (2011). ADJUST: an automatic EEG artifact detector based on the joint use of spatial and temporal features. *Psychophysiology* 48, 229–240. 20636297 10.1111/j.1469-8986.2010.01061.x

[B48] Muller J. Pentyala S. Dilger J. Pentyala S. (2016). Ketamine enantiomers in the rapid and sustained antidepressant effects. *Ther. Adv. Psychopharmacol.* 6 185–192. 10.1177/2045125316631267 27354907 PMC4910398

[B49] Murrough J. W. Yaqubi S. Sayed S. Charney D. S. (2015). Emerging drugs for the treatment of anxiety. *Expert Opin. Emerg. Drugs* 20 393–406. 10.1517/14728214.2015.1049996 26012843 PMC4869976

[B50] Muthukumaraswamy S. D. Shaw A. D. Jackson L. E. Hall J. Moran R. Saxena N. (2015). Evidence that subanesthetic doses of ketamine cause sustained disruptions of NMDA and AMPA-mediated frontoparietal connectivity in humans. *J. Neurosci.* 35 11694–11706. 10.1523/JNEUROSCI.0903-15.2015 26290246 PMC4540803

[B51] Nemeroff C. B. (2007). Prevalence and management of treatment-resistant depression. *J. Clin. Psychiatry* 68 17–25. 17640154

[B52] Olesen J. Gustavsson A. Svensson M. Wittchen H. U. Jonsson B. CDBE2010 Study Group, et al. (2012). The economic cost of brain disorders in Europe. *Eur. J. Neurol.* 19 155–162. 10.1111/j.1468-1331.2011.03590.x 22175760

[B53] Pinault D. (2008). N-methyl d-aspartate receptor antagonists ketamine and MK-801 induce wake-related aberrant γ oscillations in the rat neocortex. *Biol. Psychiatry* 63 730–735. 10.1016/j.biopsych.2007.10.006 18022604

[B54] Rivolta D. Heidegger T. Scheller B. Sauer A. Schaum M. Birkner K. (2015). Ketamine dysregulates the amplitude and connectivity of high-frequency oscillations in cortical–subcortical networks in humans: evidence from resting-state magnetoencephalography-recordings. *Schizophr. Bull.* 41 1105–1114. 10.1093/schbul/sbv051 25987642 PMC4535642

[B55] Rodriguez C. I. Kegeles L. S. Levinson A. Feng T. Marcus S. M. Vermes D. (2013). Randomized controlled crossover trial of ketamine in obsessive-compulsive disorder: proof-of-concept. *Neuropsychopharmacology* 38 2475–2483. 10.1038/npp.2013.150 23783065 PMC3799067

[B56] Roy-Byrne P. (2015). Treatment-refractory anxiety: definition, risk factors, and treatment challenges. *Dialogues Clin. Neurosci.* 17 191–206. 26246793 10.31887/DCNS.2015.17.2/proybyrne PMC4518702

[B57] Schűttler J. Stanski D. R. White P. F. Trevor A. J. Horai Y. Verotta D. (1987). Pharmacodynamic modelling of the EEG effects of ketamine and its enantiomers in man. *J. Pharmacokinet. Biopharm.* 15 241–253. 10.1007/BF01066320 3668802

[B58] Shadli S. M. Ando L. C. McIntosh J. Lodhia V. Russell B. R. Kirk I. J. (2021). Right frontal anxiolytic-sensitive EEG ‘theta’ rhythm in the stop-signal task is a theory-based anxiety disorder biomarker. *Sci. Rep.* 11:19746. 10.1038/s41598-021-99374-x 34611294 PMC8492763

[B59] Shadli S. M. Glue P. McIntosh J. McNaughton N. (2015). An improved human anxiety process biomarker: characterization of frequency band, personality and pharmacology. *Transl. Psychiatry* 5:e699. 10.1038/tp.2015.188 26670284 PMC5068587

[B60] Shadli S. M. Kawe T. Martin D. McNaughton N. Neehoff S. Glue P. (2018). Ketamine effects on EEG during therapy of treatment-resistant generalized anxiety and social anxiety. *Int. J. Neuropsychopharmacol.* 21 717–724. 10.1093/ijnp/pyy032 29718262 PMC6070106

[B61] Spielberger C. D. Gorsuch R. L. Lushene R. Vagg P. Jacobs G. (1983). *Manual for the State-Trait Anxiety Inventory.* Palo Alto, CA: Consulting Psychologists Press.

[B62] Stewart J. L. Coan J. A. Towers D. N. Allen J. J. (2014). Resting and task-elicited prefrontal EEG alpha asymmetry in depression: support for the capability model. *Psychophysiology* 51 446–455. 10.1111/psyp.12191 24611480 PMC3984363

[B63] Van Ameringen M. Patterson B. Turna J. Pipe A. Nakua H. (2017). The treatment of refractory generalized anxiety disorder. *Curr. Treat. Options Psychiatry* 4 404–417. 10.1007/s40501-017-0129-6

[B64] Wise V. McFarlane A. C. Clark C. R. Battersby M. (2011). An integrative assessment of brain and body function ‘at rest’ in panic disorder: a combined quantitative EEG/autonomic function study. *Int. J. Psychophysiol.* 79 155–165. 10.1016/j.ijpsycho.2010.10.002 20950657

[B65] Zarate C. A. Jr. Machado-Vieira R. (2017). Ketamine: translating mechanistic discoveries into the next generation of glutamate modulators for mood disorders. *Mol. Psychiatry* 22 324–327. 10.1038/mp.2016.249 28070122 PMC5641407

[B66] Zarate C. A. Jr. Mathews D. Ibrahim L. Chaves J. F. Marquardt C. Ukoh I. (2013). A randomized trial of a low-trapping nonselective N-methyl-D-aspartate channel blocker in major depression. *Biol. Psychiatry* 74 257–264. 10.1016/j.biopsych.2012.10.019 23206319 PMC3594049

[B67] Zarate C. A. Singh J. B. Carlson P. J. Brutsche N. E. Ameli R. Luckenbaugh D. A. (2006). A randomized trial of an n-methyl-d-aspartate antagonist in treatment-resistant major depression. *Arch. Gen. Psychiatry* 63 856–864. 10.1001/archpsyc.63.8.856 16894061

[B68] Zuure M. B. Hinkley L. B. Tiesinga P. H. E. Nagarajan S. S. Cohen M. X. (2020). Multiple midfrontal thetas revealed by source separation of simultaneous MEG and EEG. *J. Neurosci.* 40 7702–7713. 10.1523/JNEUROSCI.0321-20.2020 32900834 PMC7531541

